# Penicillium species as a rare isolate in tracheal granulation tissue: a case series

**DOI:** 10.1186/1752-1947-2-84

**Published:** 2008-03-17

**Authors:** Premjit S Randhawa, SA Reza Nouraei, David J Howard, Gurpreet S Sandhu, Michael A Petrou

**Affiliations:** 1 Department of Otolaryngology, Charing Cross Hospital, London, UK; 2 Royal National Throat Nose and Ear Hospital, London, UK; 3 Department of Medical Mycology, Hammersmith Hospital, London, UK

## Abstract

**Introduction:**

Granulation tissue formation is a major problem complicating the treatment of upper airway stenosis. We present two cases of recurrent tracheal granulation tissue colonisation by *Penicillium *species in patients undergoing laryngotracheal reconstructive surgery for post-intubation tracheal stenosis. We believe that although most *Penicillium *species do not cause invasive disease they can be a contributory factor to the occurrence of upper airway stenosis.

**Case presentation:**

A microbiological and mycological study of tracheal granulation tissue in two patients with recurrent laryngotracheal stenosis was carried out. *Penicillium *species was seen microscopically and cultured from tracheal granulation tissue. Neither patient grew any bacteria known to be associated with airway granulation tissue formation. Amphotericin B, itraconazole, flucytosine voriconazole and caspofungin were highly active against both isolates.

**Conclusion:**

A search for a fungal cause should form part of the investigation for recurrent tracheal granulation tissue during laryngotracheal reconstruction.

## Introduction

The commonest cause of upper airway stenosis in all age groups is post-intubation tracheal injury. This condition causes significant pulmonary morbidity and, if left untreated, may progress to life-threatening airway compromise. A major problem encountered during laryngotracheal reconstruction is the formation of airway granulation tissue.

Bacteria such as *Pseudomonas aeruginosa *and *Staphylococcus aureus *have been associated with airway granulation tissue formation [1-3], and local and systemic antibiotic prophylaxis for these organisms has been recommended [4]. However there remains a cohort of patients with recurrent airway granulation tissue, in whom no evidence of bacterial infection or foreign body reaction can be identified.

We describe two cases of biopsy-proven *Penicillium *species isolated from tracheal granulation tissue in patients with recurrent airway granulation tissue.

## Case presentation

### Case 1

A previously healthy 60-year-old male was intubated for a two-week period following myocardial infarction and as a consequence developed a 3.6 cm tracheal stenosis below the vocal cords. He underwent several microlaryngoscopy, laser and dilatation procedures to restore airway lumen, and had a soft silastic stent *in situ *to maintain luminal patency. However, he continued to have recurrent airway granulation tissue. A sample of the granulomatous tissue and the airway stent were taken and sent for microbiological and mycological investigations. No bacterial cause for the granulation tissue was identified; however, direct microscopy of the homogenised tissue showed septate hyphae pathognomonic of *Penicillium *species. In view of the recurring and florid nature of the granulation tissue, it was felt that the best long-term outcome would be an *en-bloc *tracheal resection with end-to-end anastomosis. The patient underwent the procedure successfully and has had no further problems with his airway. As he had a definitive and successful procedure, he did not require antimicrobial treatment.

### Case 2

**Figure 1:**
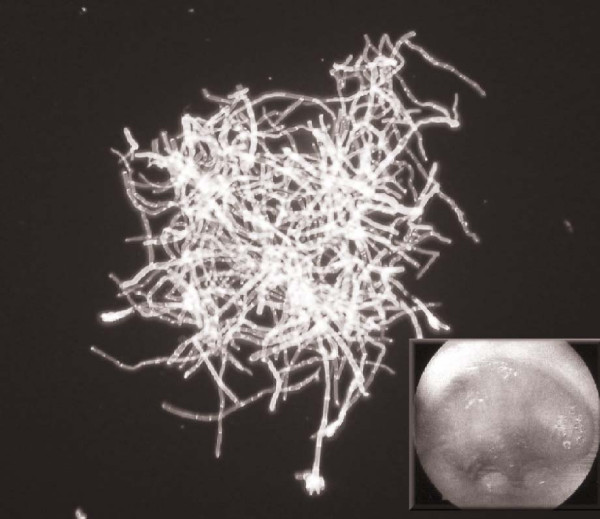
**Microscopic appearance of *Penicillium *species grown from tracheal granulation tissue**. The inset shows the endoscopic appearance of tracheal granulation tissue.

A 46-year-old male acquired a subglottic stenosis following a 10-day period of intubation after surgical clipping of a cerebral aneurysm, having acutely presenting with a subarachnoid haemorrhage. He underwent microlaryngoscopy, treatment with potassium-titanyl-phosphate (**KTP**) laser and stenting to improve his airway. Two months later he underwent repeat microlaryngoscopy and removal of the tracheal stent, at which point florid airway granulation tissue was noted (Figure 1). A sample of the granulomatous tissue and the stent were taken. Again, as in the first case, septate hyphae were seen microscopically and only *Penicillium *species were isolated. This patient subsequently underwent further microlaryngoscopy and laser therapy to the granulation tissue in his airway with significant improvement. In view of the significant improvement in his airway, antimicrobials were felt to be unnecessary.

## Method of tissue culturing

The tissue was obtained from the patients undergoing microlaryngoscopy under a general anaesthetic with an endotracheal tube in place. Biopsies were obtained from the granulomatous tissue and immediately dispatched to the department of mycology in sterile saline. The tissue was cut into smaller pieces and homogenised by drudging it onto a grid that was scarred in the middle of a sterile Petri dish with a sterile scalpel. The tissue was subsequently suspended in 1 ml of sterile distilled water. This technique has been described and used successfully by Nouraei et al [5] in their work on bacterial colonisation of airway stents.

The resulting suspension was inoculated onto blood agar aerobically and anaerobically, Maconkey agar, Neomycin blood agar anaerobically with a metronidazole disc added on the streak 2 cm away from the inoculum, chocolate agar under 10% carbon dioxide (CO_2_) and Sabouraud's dextrose agar at 30 and 37°C. The plates were incubated and were examined for growth after 2, 5 and 7 days incubation. The suspension was also treated with 20% potassium hydroxide (KOH) for 30 minutes after which a drop of calcofluor white was added and the suspension was examined using a fluorescent microscope. A slide was also prepared for Gram staining and this was examined with the aid of a light microscope using an oil immersion.

## Results

Scanty normal mouth flora was obtained from the granulation tissue of both patients and a heavy growth of *Penicillium *species was obtained only at 30°C in both cases. Following reincubation at 30°C, *Penicillium *species was grown on all of the plates without exception. The minimum inhibitory concentrations (MIC) to amphotericin B, flucytosine, fluconazole, itraconazole, voriconazole and caspofungin for both isolates were performed according to the Clinical Laboratory Standard Institute (CLSI, previously known as NCCLS) guidelines with a minor modification [6] and were incubated at 30°C until appreciable growth was achieved to be able to distinguish between growth and inhibition.

Enquiries were made to have the two isolates genotyped by molecular techniques; however, at present no such techniques are available for *Penicillium *species.

## Discussion

Airway granulation is a common and troublesome problem during laryngotracheal reconstruction. It leads to recurrent narrowing of the airway lumen and symptomatic relapse, which often can delay definitive surgical management [1,2]. Airway stents are commonly deployed in this setting to maintain luminal patency, but many patients continue to form granulation tissue, in part because of the presence of the airway stents themselves [7].

The development of granulation tissue has been associated with a number of factors notably mechanical irritation and microbial infection with bacteria such as *P. aeruginosa *and *S. aureus *[5], as well as fungi such as *Candida *species [1,2,8]. It has furthermore been shown that treatment of these infections reduces the incidence of granulation tissue formation [4], but there remains a small number of patients who continue to form airway granulation tissue with no apparent underlying cause.

In this study we isolated *Penicillium *species from two such patients, one year apart, with post-intubation tracheal stenosis and a clinical picture of recurrent airway granulation tissue associated with silastic airway stents. *Penicillium *species are ubiquitous and their spores are spread by wind and insects and are usually regarded as unimportant in terms of causing disease. Most *Penicillium *species are plant pathogens and are responsible for the spoilage of fruit and are incapable of growing at temperatures above 30°C. *Penicillium *can occasionally cause infection in humans, particularly in immunocompromised hosts, and the resulting infections are generically known as Penicilliosis. They have been isolated from patients with brain abscesses [9], necrotising oesophagitis [10], pneumonia and lung nodules [11], bone marrow [12], keratitis and conjunctivitis [13], otomycosis, endocarditis, peritonitis and urinary tract infections.

In both of our immunocompetent patients, hyphae consistent with *Penicillium *were seen on direct microscopy, where the brush-like appearance of the *penicillus *was evident. *Penicillium *species were isolated from both patients' granulation tissue (Figure 1). To the best of the authors' knowledge this has not been described in the literature previously. Both isolates were unable to grow above 35°C suggesting that neither was capable of causing deep tissue invasive disease. The granulation tissue observed could be a result of superficial growth where the temperature remained below 35°C or a result of toxic by-products produced during the growth of the *Penicillium *biofilm (Figure 1).

With the exception of fluconazole (MIC > 64 μg/ml), the isolates were inhibited by low concentrations of amphotericin B (MICs 0.012, 0.06), flucytosine (MICs 2, 0.25), itraconazole (MICs 0.01, 0.03), voriconazole (MICs 0.03, 0.02) and caspofungin (effective at 0.007 μg/ml). This suggests that these isolates are very easy to treat with appropriate antifungals, particularly the two triazoles (itraconazole and voriconazole) which are both available as oral agents. In both of our cases, the surgical removal of the granulomatous tissue has been sufficient and neither patient required antifungal treatment. However, should the granulation tissue have recurred, suggesting this was an invasive disease by an organism that *in vitro *cannot grow at body temperature but possibly is able to grow *in vivo*, which is not uncommon with some fungi, we would have opted for an oral antifungal such as itraconazole or voriconazole.

## Conclusion

We demonstrated growth of *Penicillium *species in two patients with a clinical history of recurrent airway granulation during treatment of post-intubation tracheal injury. If surgical intervention proves inadequate to eradicate the granulation, microbial causes need to be considered. We propose that a search for a fungal aetiology should be undertaken in patients with recurrent airway granulation in whom a first-line bacterial cause for the granulation cannot be identified. To this end we recommend that specimens should be cultured both at 28–30°C, as well as at 37°C, as many fungal species, including both of our isolates, do not grow above 30°C. This case illustrates that fungi that are normally considered as contaminants, such as *Penicillium *species, should not be dismissed as a possible aetiological factor to the formation of granulation tissue in the trachea.

## Competing interests

The author(s) declare that they have no competing interests.

## Authors' contributions

PSR and SARN were responsible for drafting the manuscript. MAP, DJH and GSS performed critical revision of the manuscript for important intellectual content. PSR, SARN and MAP provided administrative, technical and material support. DJH and GSS supervised the study.

## Consent

Written informed consent was obtained from both patients for publication of these case reports and accompanying images. A copy of the written consent is available for review by the Editor-in-Chief of this journal.

## References

[B1] Schmal F Fegeler W Terpe HJ Hermann W Stoll W Becker K Bacteria and granulation tissue associated with Montgomery T-tubes Laryngoscope 2003 113 1394 1400 10.1097/00005537-200308000-00024 12897565

[B2] Simoni P Wiatrak BJ Microbiology of stents in laryngotracheal reconstruction Laryngoscope 2004 114 364 366 10.1097/00005537-200402000-00034 14755219

[B3] Noppen M Pierard D Meysman M Claes I Vincken W Bacterial colonization of central airways after stenting Am J Respir Crit Care Med 1999 160 672 677 10430745 10.1164/ajrccm.160.2.9812081

[B4] Sasaki CT Horiuchi M Koss N Tracheostomy-related subglottic stenosis: bacteriologic pathogenesis Laryngoscope 1979 89 857 865 449531 10.1288/00005537-197906000-00001

[B5] Nouraei SA Petrou MA Randhawa PS Singh A Howard DJ Sandhu GS Bacterial colonization of airway stents: a promoter of granulation tissue formation following laryngotracheal reconstruction Arch Otolaryngol Head Neck Surg 2006 132 1086 1090 http://www.ncbi.nlm.nih.gov....bmed_RVAbstractPlus 10.1001/archotol.132.10.1086 17043256

[B6] Petrou MA Shanson DC Susceptibility of *Cryptococcus neoformans *by the NCCLS microdilution and Etest methods using five defined media J Antimicrob Chemother 2000 46 815 818 10.1093/jac/46.5.815 11062205

[B7] Noppen M Pierard D Meysman M Herreweghe RV Vincken W Absence of bacterial colonization of the airways after therapeutic rigid bronchoscopy without stenting Eur Respir J 2000 16 1147 1151 10.1034/j.1399-3003.2000.16f22.x 11292121

[B8] Matt BH Myer CM Harrison CJ Reising SF Cotton RT Tracheal granulation tissue. A study of bacteriology Arch Otolaryngol Head Neck Surg 1991 117 538 541 1902356 10.1001/archotol.1991.01870170084019

[B9] Noritomi DT Bub GL Beer I da Silva AS de Cleva R Gama-Rodrigues JJ Multiple brain abscesses due to *Penicillium *spp infection Rev Inst Med Trop Sao Paulo 2005 47 167 170 16021292 10.1590/s0036-46652005000300010

[B10] Hoffman M Bash E Berger SA Burke M Yust I Fatal necrotizing esophagitis due to *Penicillium chrysogenum *in a patient with acquired immunodeficiency syndrome Eur J Clin Microbiol Infect Dis 1992 11 1158 1160 10.1007/BF01961135 1291312

[B11] Liebler GA Magovern GJ Sadighi P Park SB Cushing WJ Penicillium granuloma of the lung presenting as a solitary pulmonary nodule JAMA 1997 237 671 10.1001/jama.237.7.671 576300

[B12] So CC Wong KF Bone marrow penicilliosis Br J Haematol 2002 117 777 10.1046/j.1365-2141.2002.03526.x 12060110

[B13] Miguelez S Obrador P Vila J Conjunctival infection due to *Penicillium *sp Arch Soc Esp Oftalmol 2003 78 55 57 12571777

